# Inter-City Emotional Synchrony Is Conditional on Mobility Patterns

**DOI:** 10.3390/bs12110410

**Published:** 2022-10-25

**Authors:** Karl Vachuska

**Affiliations:** Department of Sociology, University of Wisconsin-Madison, 1180 Observatory Dr., Madison, WI 53703, USA; vachuska@wisc.edu

**Keywords:** emotional contagion, twitter data, sentiment analysis

## Abstract

Recent research has attempted to document large-scale emotional contagion on online social networks. Despite emotional contagion being primarily driven by in-person mechanisms, less research has attempted to measure large-scale emotional contagion in in-person contexts. In this paper, I operationalize the temporal emotions associated with a particular city at particular points in time using sentiment analysis on Twitter data. Subsequently, I study how emotions converge between seven proximal cities in the state of Virginia, using two-way fixed effect models. I find that positive emotions tend to be synchronous between cities, but that effect is conditional on the level of contact between city residents at that period of time, as indicated by cell phone mobility data. I do not find any synchrony based on other types of emotions or general sentiment. I discourage drawing causal conclusions based on the presumed existence of several unmeasured sources of bias.

## 1. Introduction

Emotional contagion at the individual scale constitutes a well-studied phenomenon. There is clear evidence that emotional contagion is causal, and the mechanisms of the contagion have been increasingly well-understood [1,2]. When an individual is exposed to a particular emotional state, they are more likely to experience that emotional state themselves, primarily as a result of facial mimicking and behavioral observation. Some research has also found that certain emotional contagion mechanisms are chemosensory, implying that emotion can spread without direct social interaction [3].

The consequences of emotional contagion are certainly widespread but likely not well-measured. Just as the consequences of infectious disease are that more people are more easily infected with a sickness, a clear adverse consequence of emotional contagion is that more people are plagued with negative emotions. Unlike an infectious disease, however, emotional contagion may be beneficial when it constitutes the spread of positive emotions. Emotions are essential to human well-being, and sustained positive emotions are strongly associated with a number of health benefits, including “lower blood pressure, reduced risk for heart disease, healthier weight, better blood sugar levels, and longer life.” [4].

Just as a natural consequence of a disease being contagious is that it spreads across geography and large groups of people, should the same not be true for emotional contagion? Little research has been done on large-scale in-person emotional contagion. This is likely a result of the data necessary to study it historically being unavailable. For such a study to be done, one needs, at a minimum, (1) real-time emotion data on large groups of people and (2) real-time data on interpersonal contact within that large group of people. The first type of data has become available with the advent of Twitter data [5], while the second type of data has become available through the advent of cell phone mobility data [6].

In this paper, I study emotion by looking at 13,387 Twitter users in seven cities in the Hampton Roads region of Virginia during September 2021. (Although the pandemic did hugely impact mobility, studies show that mobility patterns had already returned to pre-pandemic levels by this period [7].) These users tweeted quite often, 1.7 times per day on average. Using this volume of tweets, I estimate the sentiment/emotion associated with tweets of residents of specific cities during specific two-hour periods. I treat these mean emotion levels as a proxy for the ambient sentiment/emotion among all residents of the specific city at the specific time. I subsequently utilize detailed data on cell phone mobility patterns to estimate the degree of in-person contact between residents of different cities during matching two-hour periods. By combining these data sources, I study how synchrony in emotion between residents of different cities is dependent on the level of contact between them. I find that ambient emotions in a specific city tend to be predicted by preceding ambient emotions in other cities, but this effect is only significant conditional on the level of contact between residents. 

Furthermore, this relationship only appears to hold true for positive emotions. Daily patterns and variations in the volume of activity on Twitter cannot explain this relationship, implying that synchrony in emotions between cities is a consequence of the level of contact between them. These results may provide suggestive evidence of a measurable, large-scale emotional contagion, but as there are multiple sources of bias that are unaccounted for, I strongly discourage causal interpretation in the absence of further research.

### 1.1. Emotion and Emotional Contagion

Emotional contagion has been defined as “the tendency to automatically mimic and synchronize expressions, vocalizations, postures, and movements with those of another person and, consequently, to converge emotionally” [1]. Multiple mechanisms have been implicated in emotional contagion.

Facial expressions are an essential component of emotional contagion. When two individuals interact, they tend to mimic each other’s facial expressions [2]. Theories of social appraisal, in turn, suggest that how individuals view an event or interaction is a product of how others view that event or interaction. Thus, if an individual sees another individual portraying a facial expression associated with a particular emotion, they not only will mimic those facial expressions but will likely adopt that emotional perspective about the event/interaction occurring. Even in the absence of social appraisal, an individual may “transmit” a specific emotion to a third individual through facial mimicking.

Physiologically, emotional contagion can operate through the convergence of autonomic nervous system responses and neural representations. Research has linked specific emotions to specific arousal states [2], again suggesting a strong specificity in emotional contagion. Research has also suggested that neurological reactions constitute an additional mechanism of emotional contagion. 

Beyond contagious emotions, physiological states that may be linked to emotion may also be contagious. Specifically, stress and cortisol are transmitted easily from person to person [8,9]. Distinct from simple emotional contagion, research has found that stress can spread through chemosensory cues, implying that stress can spread independently from any social interaction. Stress, in turn, is closely associated with various emotional states, such as anger [3]. The spread of stress has been documented to travel with people between different contexts and locations. Bolger et al. [10] showed that spouses spread stress between one another and bring stress to and from home and work with them.

While much research has examined emotional contagion on a positive-negative scale, more refined research has documented that more specific types of emotions tend to be contagious. For example, Olszanowski et al. [11] exposed subjects to videos of individuals displaying either sadness or anger. While sadness and anger are both generally considered negative, subjects’ exposure to the emotions resulted in specific mimicking and specific self-reported feelings. This research suggests that reducing emotion to a sentiment continuum may overgeneralize the emotional state. Additional research suggests that contagion is emotion-specific, and specific emotions transmit to other distinct emotions minimally. Wild et al. [12] found that exposing subjects to happy faces tended to result in subjects experiencing happy affect, exposing subjects to sad faces tended to result in subjects experiencing sad affect, and exposure to either had a less consistent effect on distinct emotions such as anger, disgust, surprise, fear, and pleasure.

More recent work has documented the transmission of emotion at larger scales. Coviello et al. [13] documented emotional contagion in an online social network. Their work utilized an instrumental variable approach, showing that rain near where an individual lived predicted negative emotion in their Facebook posts, which predicted negative emotion in their friends’ Facebook posts, even ones that did not experience rain. The authors argued that online social networks magnify global emotional synchrony by allowing emotion to spread quickly across vast geographical distances. Other research has provided more compelling evidence of the contagion using experimental methods. Kramer et al. [14] modified Facebook users’ news feed to show either more positive or negative content. The authors found that the likelihood of a user’s post being either positive or negative was directly manipulated by the amount of positive or negative posts they were exposed to. These results notably demonstrate that in-person contact is unnecessary for emotional contagion. Notably, the effect sizes of the experiment were acknowledged as being very small.

In this article, I hope to build on past work that has measured emotion using online social media data by adding real-world human mobility data as a measure of the connections and relations through which emotion may spread. While past research has focused on how emotions spread between online social media users who are presumed to strictly be connected through digital means, I believe in-person, aggregate measures of human contact may be a more useful pathway with which to analyze diffusion. Research on emotional contagion suggests that in-person interaction components, such as witnessing facial expressions, behavioral observation, and exposure to chemosensory signals, are the dominant mechanisms of emotional contagion. Thus, little theory suggests that a digital emotional contagion would be substantial. On the other hand, an in-person large-scale emotional contagion should be far more sizeable.

### 1.2. General Hypotheses

While much work has studied emotional contagion in single settings, less work has looked at it as a large-scale in-person process [15]. Often, traditional epidemiological models analyze and predict how and when individuals shift between three states: Susceptible, Infected and Recovered. The probability that individuals will shift between these states determines the predicted scale and speed of disease spread. In the case of applying emotional contagion to this model, I lack the detailed, fine-grained data to simulate large-scale person-to-person spread accurately. So instead, I will focus on theorizing based on the two coarse attributes of contagion that make the most considerable difference: the probability of infection when exposed and the duration of infection prior to recovery.

The strength of emotional contagion has been frequently investigated. Research strongly suggests that emotions are diminished in intensity through contagion rather than perfectly replicated or amplified. Wild et al. [12] demonstrated the importance of expression strength in evoking emotions. Notably, they found that weaker happy and sad expressions evoked weaker levels of happiness and sadness. Additionally, emotional contagion is not uniform, and there is wide variation in the level of emotion evoked. Not everyone becomes happier when exposed to happy expressions, even when that exposed level of happiness is very high. Presumably, this diminishing of intensity and the variation in effect will lead emotional contagion to weaken very quickly after just a few degrees of contact.

The duration of emotional states varies considerably depending on the emotion considered [16]. Research has found that most emotions last between one and six hours. Some emotions, such as shame, relief, and disgust, tend to last less than an hour, while anger, enthusiasm, and sadness have been found to last for 24 h or longer. More important than the unique emotion, however, perceived event importance and rumination explain most of the variability in how long emotions last. A realistic model of emotional contagion assumes no overt awareness of the contagion effect, thus implying no perceived “event” was tied to the emotional transmission, subsequently minimizing the likelihood of substantial, if any, perceived event importance and rumination. This suggests that the duration of infection for an emotional contagion should be fairly short-lived, likely no more than a few hours.

Ultimately, two variables are important to consider in predicting how emotion might spread among large groups of people. First, while emotions are strongly contagious, emotion is not a discrete concept, and a contagion effect cannot be expected to uniformly affect individuals’ emotional states and likely cannot be measurable over many degrees of contact. Second, emotional states that result from emotional contagion should be short-lived. Taken together, these two attributes inform how emotional contagion on a large scale should appear. Large-scale analyses of emotional contagion should focus on *aggregate or averaged* levels of emotional state. Strictly looking for extreme cases of emotion is inappropriate as emotion is fluid and spreads variably but generally diminishes in intensity as it spreads. Additionally, large-scale emotional contagion is likely diluted quickly and cannot be expected to be measured over many steps. Lastly, given assumptions regarding how long emotions that result from emotional contagion last, emotional contagion is likely best measured within a few hours of the initial exposure.

### 1.3. Causal Peer Effects and Subsequent Limitations

Measuring contagion, otherwise known as causal peer effects, is an incredibly challenging problem in causal inference and statistics. A notable example, Christakis and Fowler [17] argued they had evidence of obesity spreading as a contagion through a social network based on an individual’s weight gain being significantly predicted by the weight gain of friends, siblings, and spouses. However, their paper was subsequently heavily criticized for utilizing a methodological approach from which inference of causal peer effects could not be plausibly drawn. Among the greatest criticisms, researchers have pointed out that Christakis and Fowler [17] had no way of controlling for confounding environmental factors, which certainly affect the likelihood of weight gain. Indeed, a subsequent study by Cohen-Cole and Fletcher [18] found that environmental factors could entirely explain the “contagiousness” of weight gain.

Various methods have been utilized to draw a stronger degree of causal inference in peer effects. Among the most popular is the instrumental variable approach. In this approach, researchers utilize variables that are predictive (in both conceptual and operational terms) of a specific target individual experiencing a particular outcome but that are theoretically unrelated to an individual’s friends experiencing the same outcome, other than the causal path that runs through a contagion effect. An example of this application can be found in Aral and Nicolaides [19], where the authors examine data from an online exercise-focused social network where individuals can document and share information on their runs. The authors argued theoretically and demonstrated statistically that local weather conditions (specifically rain) are a relatively strong predictor of whether or not someone runs. Since the social network is online, an individual may be connected to others on the site that live in geographically distant locations (where different weather conditions are occurring), and the authors used rain as an instrumental variable, finding that running is significantly “contagious”.

A fundamental property of drawing causal inference in peer effects is getting the temporal ordering right. Fully synchronous effects say nothing about causal peer effects as they can easily result from a time-varying confounding mechanism, such as exposure to a common environmental factor that induces the specific outcome. Additionally, in many cases, theory suggests the contagion effect has some delay or that the likelihood of the contagion effect is a product of the duration of exposure, which would also suggest a delay. Thus, a fully synchronous effect in many cases would not indicate a causal peer effect. On the other hand, somewhat delayed effects of the theoretically aligning duration with adequate controls for time-varying covariates provide a more substantial degree of causal inference.

In this work, I use a unique approach for attempting to draw causal inference in peer effects. Unlike much data involved in causal peer effects, connections and contact operationalized from mobility data are highly dynamic. In effect, this allows one to estimate and draw from counterfactual scenarios of if contact had occurred to a lesser extent. Since emotional contagion requires in-person contact to spread and the level of in-person contact should theoretically predict the level of contagion, in the assumption of no unobserved confounding, the level of contact between residents of different cities should only predict the level of correlation in emotion if emotional contagion is present. Unfortunately, as I will explain, there are confounding variables that are unaccounted for in my statistical models.

While I caution against drawing strong causal inferences from this article, I do attempt to demonstrate causal inference to the greatest degree the data makes possible. In terms of fitting my methods into the context of causal peer effects studies, I must acknowledge the limitations of my methodology. Regarding the actual data, it is possible that tweets are poor proxies of individuals’ actual emotions/sentiments. The sentiment associated with individuals’ online activity has been argued to be strongly influenced by other activity on the social network. While I do not have the data to control for sentiment spread between social network users directly, I do demonstrate that my findings are robust to controlling on the level of activity on Twitter in the immediate geographical area, a logical proxy of how influential Twitter activity is on users at the specific time. In either case, emotions/sentiments extracted using Natural Language Processing may not accurately reflect the real-world emotions individuals are experiencing. 

In terms of the statistical methods for drawing causal inference, this work has two notable shortfalls. First, the application of mobility data as a real-time measure of contact between groups of people may introduce shared-exposure bias into the estimated effect [20]. Shared-exposure bias suggests causal peer effects may be a noncausal artifact of shared exposure to a common environment. In the case of mobility patterns, individuals are physically exposed to more similar environments, which may induce a shared emotional state.

A second, albeit less likely, source of bias is homophily bias. Potentially, people are driven together or to the same places because of their emotional state. For example, people who are feeling energetic and in a good mood may go to a recreational center to exercise, a decision they may not have made if they were feeling sad. While this source of bias seems less likely, the scale of its impact is unknown, and thus it remains a critical limitation that needs to be acknowledged.

Because of this shared exposure and homophily bias, I do not make causal claims regarding these results. Instead, I make predictive claims and emphasize the importance of real-time mobility patterns in predicting synchrony in emotion. Unlike other topics in causal peer effects, mobility patterns are highly dynamic and constantly varying, thus giving mere predictive power greater value.

## 2. Materials and Methods

### 2.1. Twitter Data

**Figure 1:**
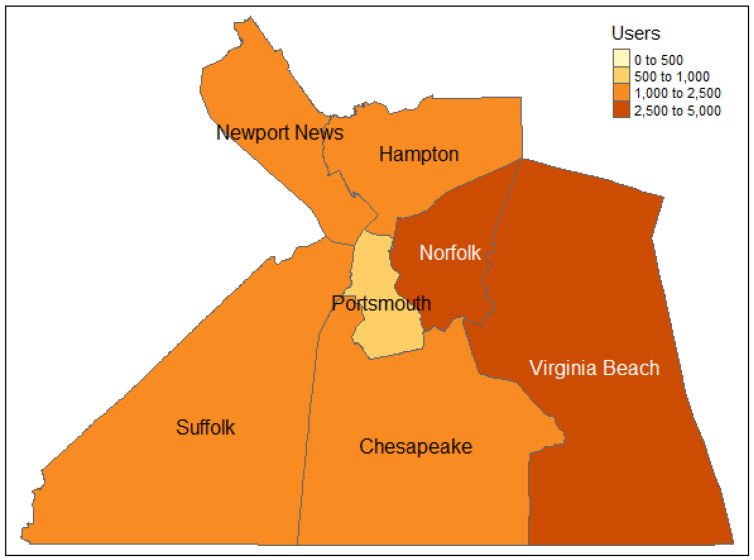
Distribution of Twitter users by city.

Data for this project comes from the Twitter API. Specifically, I request all tweets geocoded to within 30 miles of Norfolk, VA, between 1 September and 30 September 2021. User profiles are attached to the tweets and examined to geocode users to specific cities. Users are assigned to the city self-identified in their profile. All tweets and users that list a location outside of seven main cities: Suffolk, Virginia Beach, Newport News, Norfolk, Hampton, Chesapeake, and Portsmouth, are excluded. The motive for choosing Hampton Roads as a region of study is the unique urban structure of the area. The Hampton Roads lacks as clear of a principal city compared to other U.S. metropolitan areas. Instead, the seven largest cities all have between 90,000 and 450,000 residents and are located directly adjacent to one another. Subsequently, the level of interaction between residents of the seven cities is high, and each city has a substantial population from which to take a sample. As a social norm, Twitter users tend to identify where they live in their profile by listing the name of their city. Thus, the city-level is the smallest scale at which it is convenient to study Twitter users. Figure 1 shows the boundaries of the seven cities and the number of Twitter users associated with each city in this analysis [21].

The Twitter dataset contains 678,941 tweets by 13,387 users. Each tweet is assigned to the two-hour period it falls into. Using the Syuzhet package in R [22], I obtain a sentiment score and check for the presence of 10 NRC sentiments: “anger”, “anticipation”, “disgust”, “fear”, “joy”, “sadness”, “surprise”, “trust”, “negative”, and “positive”. While sentiment scores are on a continuous scale, NRC sentiment presence is measured as an integer, denoting the number of words in the tweet associated with the NRC sentiment. Subsequently, I code tweets dichotomously depending on whether or not at least one word is associated with each specific NRC sentiment.

Because emotional sentiment may vary between users and users may tend to tweet at different times, I standardize each sentiment measure for each user’s tweets to ensure that variation in who tweets when should not drive spurious relations in time. Subsequently, for all emotion/sentiment measures, I estimate “ambient” emotion/sentiment measures for each city in each two-hour period in the month by individually averaging these user-standardized emotion/sentiment measures across all tweets made by sets of residents in the two-hour period.

Although there may be some disconnect between how users portray themselves online and their real offline emotional state, research has found the two have, in aggregate, a very meaningful relationship [23,24,25,26]. Subsequently, I believe sentiment analysis of social media data can serve as a proxy of offline emotional state. Sentiment analysis can be an especially powerful data reduction tool, given the scale of data involved here makes it essentially impossible to perform a nonautomated qualitative analysis.

### 2.2. Mobility Data

Mobility data for this project comes from SafeGraph’s “Patterns” dataset. Specific mobility patterns for September 2021 for the Virginia Beach-Newport News metropolitan area are obtained. Points of interest are geocoded to Virginia cities. Origin neighborhoods are additionally geocoded to Virginia cities. While points of interest do not have hourly visitor data, I can infer hourly visitor data by combining data on mobility patterns for the entire month, daily data, and hourly data aggregated across the entire month. 

Specifically, for a specific hour at a specific point of interest, I estimate the level of contact between residents of two different cities through the following equation:

$$C_{ijklm}=p_{im}*p_{jm}*V_{km}*P_{lm}$$

where $p_{im}$ represents the proportion of visitors to point of interest *m* that reside in city *i*, $V_{km}$ represents the total number of visitors to point of interest *m* during hour *k* across the entire month of September 2021, and $P_{lm}$ represents the total proportion of visitors to point of interest *m* that were made on day *l.*

For each pair of cities, I sum the level of contact across all points of interest in order to come up with unique aggregate levels of contact for each pair of cities for each one-hour period in September 2021. I then sum the levels of contact for adjacent one-hour periods together to obtain new estimates of the level of contact for two-hour periods.

### 2.3. Combining Twitter and Mobility Data

I combine Twitter and mobility data to create a mean degree-weighted score for each emotion/sentiment measure. This effectively estimates the mean emotion/sentiment score across the six other cities, weighting each city by the level of contact residents of the target city have with residents of each other city during that specific period. I additionally estimate the total level of contact between residents of the target cities and all other cities in each two-hour period by summing the six measures of contact with each other city.

In total, I create 11 longitudinal emotion/sentiment measures for all seven cities for every two-hour period that had at least 50 tweets. The first is a general sentiment score, while the other 10 are averaged presence of certain emotions/sentiments: “anger”, “anticipation”, “disgust”, “fear”, “joy”, “sadness”, “surprise”, “trust”, “negative”, and “positive”.

While I analyze general sentiment as a single phenomenon, I aggregate the 10 specific emotions into a long-form dataset for a cumulative analysis. The secondary dataset is arranged such that each observation constitutes a unique combination of city, two-hour period, and emotion type. The dependent variable is the mean scaled presence of the specific emotion for specific city residents in the specific two-hour period. 

I subsequently analyze all ten emotions in aggregate, in separation, and in groups. The groupings are organized as follows. Positive emotions are operationalized as “joy”, “trust”, and “positive”. Negative emotions are operationalized as “anger”, “disgust”, “sadness”, and “negative”. Lastly, tense emotions are operationalized as “anticipation”, “fear”, and “surprise”. Table 1 displays summary statistics for all variables. 

### 2.4. Methods

I utilize a fixed-effects model to estimate the mobility-conditional effect of emotion/sentiment contagion. The central intuition behind the model is that while emotion/sentiment may vary naturally between time and place, the level of mobility should only predict the level of association between the emotion/sentiment of different cities’ residents if an emotional contagion exists. I additionally control on the lagged emotion/sentiment score for the specific city in the previous two-hour period.

For a specific sentiment/emotion measure *S,* my preferred specification can be written as:

(1) $$S_{it}=\beta_{1}S_{it-1}+\beta_{2}MED_{it-1}+\beta_{3}CONT_{it-1}+\beta_{4}MED_{it-1}*CONT_{it-1}+\Delta_{t}+\varepsilon$$

where $S_{it}$ constitutes the measure in city *i* at time *t* (Where time *t*, is one of 360 unique 2-h periods in the month), $MED_{it-1}$ constitutes the mean degree-weighted emotion for city *i* at time *t* − 1, $CONT_{it-1}$ represents the aggregate level of contact between residents of city *i* and all other cities at time *t* − 1, ${∆}_{t}$ represent hour-specific fixed-effects, and ε represents an error term.

The coefficient $\beta_{4}$ here provides the causal estimate of the effect of mobility on the contagiousness of the sentiment. More specifically, a positive significant coefficient here indicates that the mean degree of emotion in contact predicts emotion in the target city but that that effect is conditional on the level of contact. Given theory suggesting that emotions may spread through contact and that common environments may induce common emotional states, I hypothesize that the coefficient $\beta_{4}$ will be positive.

In aggregating multiple emotions into the same model, I estimate the same model, but instead of hour-specific fixed effects, I include hour-emotion-specific fixed effects.

As a first robustness check, I additionally estimate a second specification, which includes fixed effects on the specific combinations of hour of the day and city. This specification controls for the fact that residents of certain cities may inherently feel certain ways at specific times of the day, and residents of certain cities may also exhibit certain mobility patterns at specific times of the day. Such a phenomenon could easily drive a spurious relationship between mobility and sentiment, given that mobility patterns are fairly consistent at the hour level from day to day.

As a second robustness check, I also estimate a third specification that includes a control for the volume of Twitter activity in the specific city at the specific point in time. I operationalize this measure as the logged number of tweets by residents of the specific city recorded in the specific two-hour period, mean-standardized for each city. This robustness check helps ensure that the dissemination of specific messages across Twitter are not inducing synchrony in measured emotion.

## 3. Results

### 3.1. General Sentiment

The results for my first set of models are shown in Table 2. The variable estimated here is the mean sentiment score of a tweet for a specific city and hour, standardized to the user. These results ultimately show no evidence of general sentiment being more synchronized during times of greater contact. As a reflection on this result, it is important to recognize sentiment scores have often been considered an overreduction of data. Indeed, analysis using NRC-sentiment has been demonstrated to perform more closely to human coders in categorizing the general sentiment of text compared to sentiment scores themselves [27]. A high sentiment score does not indicate if joy or trust is present, while similarly, a low sentiment score does not indicate if anger, disgust, or sadness is present in the text. Indeed, this overgeneralization of emotional states does not align strongly with emotional contagion theory. After all, emotional contagion is not the same thing as sentiment contagion.

### 3.2. Emotions

Next, I look at predicting the level of *emotional* states of tweets for residents in specific cities at specific two-hour periods. Table 3 displays these results. All models include time-emotion fixed effects. Model 1 shows that the level of emotion in the prior period is a significant predictor of emotion in the current period. The degree of emotion is not a significant predictor, however. Distinctly, model 2 and 3 demonstrates that the focal interaction between mobility contact level and mean degree of emotion is a significant predictor, with *p* < 0.01. Model 4 includes hour-of-the-day-city-emotion specific fixed effects, demonstrating the robustness of the focal interaction and showing that everyday patterns are not causing this result. Finally, model 5 includes controls for the volume of Twitter activity in the specific city in the specific time period. The fact that the focal interaction is robust to this control suggests that increased online activity is not driving the finding.

### 3.3. Specific Sentiments/Emotions

Table 4 shows estimated models for each emotion. Surprisingly, the focal interaction is not statistically significant in any model. For two emotions, “joy” and “negative”, the focal interaction is marginally significant at *p* < 0.1. While these results are somewhat surprising, a lack of significance makes some sense given the heavily reduced sample size involved in studying each individual emotion. In the following analysis, I group emotions into larger sets.

### 3.4. Sentiment/Emotion Groupings

Table 5 depicts models for the “positive” emotion grouping. The positive emotion grouping is made up of 3 emotions: “joy”, “trust” and “positive”. All models notably show the significance of the lag term, indicating that positive emotional states have meaningful spatiotemporal variation. Models 2–5 additionally all indicate the statistical significance (*p* < 0.05) of the focal interaction term. This means the focal interaction term is not only a significant predictor in the preferred specification but is robust to controls for everyday patterns and Twitter activity volume. These findings suggest that positive emotions are likely to be synchronous during high levels of contact. This aligns with recent research on the contagiousness of emotions on Twitter, which found that the likelihood of users adopting positive emotions in their tweets based on the content of their Twitter feed was far more likely than the adoption of negative emotions [28]. In-person data additionally suggest that positive emotions are more likely to be contagious [29].

Table 6 depicts models for the “negative” emotion grouping. The negative emotion grouping is made up of 4 emotions: “anger”, “disgust”, “sadness” and “negative”. Remarkably, the focal interaction is not significant in any of the models, and the lagged dependent variable is not either. These results indicate that synchrony in negative emotions does not increase during high levels of contact and that no sustained spatiotemporal variation in negative emotions is present. These results are mixed in their alignment with past research. While some research does suggest that positive emotions are more contagious than negative emotions [29], research still finds that negative emotions are fairly contagious [11].

Table 7 depicts models for the “tense” emotion grouping. The tense emotion grouping is made up of 3 emotions: “anticipation”, “fear” and “surprise”. Similar to the negative emotion grouping, neither the focal interaction nor the lagged dependent variable is significant in any model. These results somewhat align with past research, which has documented an unclear relationship between emotional exposure and the evoking of “surprise”. Indeed, Lundquist [30] found in an experiment that among six emotional states tested, surprise induced the smallest effect on the *levator labii* and *corrugator supercilia*, two facial muscles frequently implicated in the facial mimicking component of emotional contagion.

## 4. Discussion

In this article, I have investigated large-scale emotion contagion using Twitter data as a proxy for the emotions people are experiencing. I combined these Tweets with cell phone mobility data to measure when and how contact between residents of different cities relates to the synchrony in emotion they experience.

Past research has attempted to measure large-scale emotional contagion, but has mainly examined the spread within social media platforms, rather than in in-person contexts. While evidence has been consistently documented of small emotional contagion effects on social media platforms, past research would suggest the mechanisms through which a digital emotional contagion operates are far less influential than in-person mechanisms. Thus, this work contributes to the body of literature by suggesting that in-person mechanisms may contribute to the emotions measured on social media platforms. Future research studying emotion on social media platforms should bear in mind the mechanisms through which emotion spreads between users may operate outside of the platform entirely, as the theory and results here suggest.

While this work was designed as an approach for measuring large-scale emotional contagion, I strongly discourage drawing causal conclusions. Although past work indicates that emotions are highly contagious between individuals in specific settings, the findings here do not imply that spread is measurable across large groups of people in everyday contexts. It is reasonably possible for the relation between contact levels and emotional synchrony to be a spurious byproduct of emotion-inducing stimuli in shared environments. Even without drawing causal inference from this work, the results here are still beneficial in predicting acute changes in sentiment across geographical areas. Such a finding is uniquely crucial given the highly dynamic nature of human mobility and emotion.

This paper lays a blueprint for exploring emotional contagion in the real world on a large scale. However, there are substantial caveats to interpreting these results, and future research should focus on improving this blueprint. Notably, this work only looked at seven cities in a single region of the United States. A greater understanding of large-scale emotional contagion would benefit from reproducing this approach across other cities and regions. Future research should also attempt to find approaches and models to produce stronger degrees of causal inference. As acknowledged, this approach cannot accurately measure inter-city emotional contagion because of two critical sources of bias: shared-exposure bias and homophily bias. Identifying mobility patterns that are affected by exogenous events may be a potential method for developing a quasi-experimental approach that is able to better reduce unmeasured sources of bias.

## 5. Conclusions

The results of these analyses reveal two important findings. First, in aggregate, the tendency for specific emotions to beget specific emotions between residents of different cities appears to be conditional on the degree of contact between residents of those cities. Second, the set of emotions for which this is true specifically appears to be positive emotions. I find no evidence that such a finding holds for general sentiment, negative emotions, or tense emotions. I also cannot identify individual emotions for which this finding is true. Despite taking advantage of rich data, these models exclude several unmeasured sources of bias, so I discourage drawing causal conclusions from these findings.

## Figures and Tables

**Table 1:** Summary Statistics.

Variable	Mean	Sd	Min	25%	75%	Max
Sentiment	0	0.103	−0.844	−0.054	0.054	0.649
Mean degree of sentiment	0	0.051	−0.219	−0.03	0.032	0.287
Mean degree of anger	0	0.05	−0.191	−0.031	0.025	0.192
Mean degree of anticipation	0	0.054	−0.234	−0.031	0.03	0.299
Mean degree of disgust	0	0.049	−0.219	−0.029	0.025	0.252
Mean degree of fear	0	0.052	−0.187	−0.032	0.026	0.284
Mean degree of joy	0	0.055	−0.217	−0.031	0.034	0.41
Mean degree of sadness	0	0.05	−0.187	−0.028	0.026	0.292
Mean degree of surprise	0	0.054	−0.203	−0.031	0.03	0.342
Mean degree of trust	0	0.058	−0.228	−0.037	0.035	0.302
Mean degree of negative	0	0.052	−0.219	−0.031	0.029	0.258
Mean degree of positive	0	0.06	−0.216	−0.036	0.037	0.463
Anger	0	0.105	−0.526	−0.056	0.047	0.678
Anticipation	0	0.107	−0.482	−0.055	0.056	1.313
Disgust	0	0.106	−0.411	−0.056	0.049	0.746
Fear	0	0.109	−0.456	−0.057	0.05	0.961
Joy	0	0.109	−0.595	−0.055	0.057	1.014
Sadness	0	0.104	−0.506	−0.054	0.051	0.848
Surprise	0	0.107	−0.482	−0.054	0.052	1.01
Trust	0	0.108	−0.555	−0.062	0.058	0.574
Negative	0	0.108	−0.548	−0.057	0.053	1.037
Positive	0	0.111	−0.622	−0.061	0.06	0.864
Mobility contact level	0	0.999	−1.378	−1.065	0.826	2.065
Tweet volume	0	0.999	−4.06	−0.537	0.706	1.861

**Table 2:** General sentiment models.

	Model 1	Model 2	Model 3	Model 4	Model 5
Lagged dependent variable	0.058 *	0.056 *	0.057 *	0.065 *	0.058 *
	(0.026)	(0.024)	(0.026)	(0.027)	(0.026)
Mean degree of emotion	0.013		0.007	0.019	0.009
	(0.068)		(0.068)	(0.067)	(0.068)
Mobility contact level			0.000	0.025	0.000
			(0.041)	(0.051)	(0.041)
Tweet volume					0.003
					(0.004)
Mobility contact level X Mean degree of emotion		0.091	0.090	0.087	0.090
		(0.070)	(0.070)	(0.070)	(0.070)
Tweet volume X Mean degree of emotion					−0.004
					(0.068)
Hour Fixed Effects	X	X	X	X	X
City-hour-of-day Fixed Effects				X	
N	2256	2256	2256	2256	2256
AIC	−4614.212	−4616.667	−4612.683	−4512.743	−4609.384
BIC	−2548.805	−2551.260	−2535.833	−1961.021	−2521.091
Pseudo R2	0.093	0.094	0.093	0.079	0.092

* *p* < 0.05.

**Table 3:** Emotion models.

	Model 1	Model 2	Model 3	Model 4	Model 5
Lagged dependent variable	0.021 **	0.017 *	0.020 **	0.022 **	0.019 **
	(0.008)	(0.007)	(0.008)	(0.008)	(0.008)
Mean degree of emotion	0.023		0.017	0.031	0.017
	(0.021)		(0.021)	(0.022)	(0.021)
Mobility contact level			−0.020	−0.069 ***	−0.020
			(0.014)	(0.018)	(0.014)
Tweet volume					0.001
					(0.001)
Mobility contact level X Mean degree of emotion		0.064 **	0.063 **	0.056 **	0.063 **
		(0.020)	(0.020)	(0.020)	(0.020)
Tweet volume X Mean degree of emotion					−0.011
					(0.020)
Emotion-Hour Fixed Effects	X	X	X	X	X
Emotion-City-hour-of-day				X	
N	22,560	22,560	22,560	22,560	22,560
AIC	−45,171.041	−45,183.134	−45,182.225	−44,388.637	−45,180.081
BIC	−16,349.071	−16,361.164	−16,344.208	−8818.539	−16,326.015
Pseudo R2	0.092	0.092	0.092	0.087	0.092

*** *p* < 0.001; ** *p* < 0.01; * *p* < 0.05.

**Table 4:** Specific emotion models.

	Anger	Anticipation	Disgust	Fear	Joy	Sadness	Surprise	Trust	Negative	Positive
Lagged dependent variable	−0.001	0.021	0.024	0.016	0.036	0.016	0.017	0.023	0.007	0.037
	(0.023)	(0.023)	(0.023)	(0.026)	(0.024)	(0.025)	(0.024)	(0.023)	(0.024)	(0.024)
Mean degree of emotion	−0.049	−0.024	0.019	0.004	0.003	0.075	0.040	0.011	−0.019	0.097
	(0.065)	(0.068)	(0.068)	(0.078)	(0.062)	(0.073)	(0.057)	(0.067)	(0.065)	(0.062)
Mobility contact level	−0.031	0.011	−0.035	−0.018	−0.052	0.008	−0.018	−0.036	−0.017	−0.011
	(0.045)	(0.041)	(0.046)	(0.048)	(0.045)	(0.044)	(0.045)	(0.045)	(0.046)	(0.044)
Mobility contact level X Mean degree of emotion	0.058	0.030	0.024	0.044	0.106	0.043	0.067	0.069	0.106	0.080
	(0.059)	(0.066)	(0.071)	(0.062)	(0.060)	(0.071)	(0.058)	(0.060)	(0.061)	(0.062)
N	2256	2256	2256	2256	2256	2256	2256	2256	2256	2256
AIC	−4467.339	−4647.499	−4451.656	−4282.475	−4574.188	−4572.052	−4733.688	−4525.899	−4463.899	−4434.628
BIC	−2390.489	−2570.650	−2374.807	−2205.625	−2497.338	−2495.202	−2656.838	−2449.049	−2387.050	−2357.778
Pseudo R2	0.046	0.126	0.027	0.070	0.114	0.044	0.109	0.148	0.073	0.143

**Table 5:** Positive emotion models.

	Model 1	Model 2	Model 3	Model 4	Model 5
Lagged dependent variable	0.033 *	0.027 *	0.032 *	0.033 *	0.031 *
	(0.014)	(0.013)	(0.014)	(0.014)	(0.014)
Mean degree of emotion	0.045		0.038	0.050	0.040
	(0.036)		(0.037)	(0.039)	(0.037)
Mobility contact level			−0.033	−0.084 **	−0.032
			(0.026)	(0.032)	(0.026)
Tweet volume					0.003
					(0.003)
Mobility contact level X Mean degree of emotion		0.086 *	0.083 *	0.076 *	0.087 *
		(0.035)	(0.035)	(0.036)	(0.035)
Tweet volume X Mean degree of emotion					−0.067 *
					(0.033)
Emotion-Hour Fixed Effects	X	X	X	X	X
Emotion-City-hour-of-day				X	
N	6768	6768	6768	6768	6768
AIC	−13,541.105	−13,546.723	−13,545.836	−13,285.110	−13,549.520
BIC	−6182.368	−6187.986	−6173.458	−4200.922	−6163.503
Pseudo R2	0.135	0.136	0.136	0.128	0.137

** *p* < 0.01; * *p* < 0.05.

**Table 6:** Negative emotion models.

	Model 1	Model 2	Model 3	Model 4	Model 5
Lagged dependent variable	0.012	0.011	0.012	0.015	0.012
	(0.012)	(0.011)	(0.012)	(0.012)	(0.012)
Mean degree of emotion	0.014		0.007	0.016	0.005
	(0.033)		(0.033)	(0.034)	(0.034)
Mobility contact level			−0.019	−0.062 *	−0.019
			(0.023)	(0.029)	(0.023)
Tweet volume					0.000
					(0.002)
Mobility contact level X Mean degree of emotion		0.059	0.059	0.054	0.057
		(0.032)	(0.032)	(0.034)	(0.032)
Tweet volume X Mean degree of emotion					0.039
					(0.034)
Emotion-Hour Fixed Effects	X	X	X	X	X
Emotion-City-hour-of-day				X	
N	9024	9024	9024	9024	9024
AIC	−17,971.212	−17,975.666	−17,972.520	−17,619.326	−17,970.653
BIC	−7750.422	−7754.875	−7737.514	−5003.260	−7721.432
Pseudo R2	0.048	0.048	0.048	0.039	0.048

* *p* < 0.05.

**Table 7:** Tense emotion models.

	Model 1	Model 2	Model 3	Model 4	Model 5
Lagged dependent variable	0.020	0.017	0.018	0.021	0.018
	(0.014)	(0.013)	(0.014)	(0.014)	(0.014)
Mean degree of emotion	0.011		0.007	0.029	0.008
	(0.039)		(0.039)	(0.040)	(0.040)
Mobility contact level			−0.008	−0.063	−0.008
			(0.026)	(0.034)	(0.026)
Tweet volume					0.002
					(0.003)
Mobility contact level X Mean degree of emotion		0.047	0.046	0.039	0.046
		(0.035)	(0.036)	(0.036)	(0.035)
Tweet volume X Mean degree of emotion					−0.011
					(0.039)
Emotion-Hour Fixed Effects	X	X	X	X	X
Emotion-City-hour-of-day				X	
N	6768	6768	6768	6768	6768
AIC	−13,654.161	−13,656.227	−13,652.398	−13,478.003	−13,649.700
BIC	−6295.423	−6297.489	−6280.020	−4393.815	−6263.683
Pseudo R2	0.101	0.102	0.101	0.104	0.101

## Data Availability

Restrictions apply to the availability of these data. Data was obtained from SafeGraph, whose terms of service prevent the sharing of their data.
